# Patient safety in the ‘Room of Horrors’ simulation: a multi-method study of student, novice, and experienced nurses

**DOI:** 10.1186/s12912-025-03700-x

**Published:** 2025-08-08

**Authors:** Seung Eun Lee, Hyun Joo Lee, V. Susan Dahinten, Won Jin Seo, Heejin Lim, Hanjoe Kim

**Affiliations:** 1 College of Nursing, Mo-Im KIM Nursing Research Institute, Yonsei University, 50-1 Yonsei-ro, Seodaemun-gu, Seoul, 03722 South Korea; 2 School of Nursing, University of British Columbia, T201-2211 Wesbrook Mall, Vancouver, BC V6T 2B5 Canada; 3 College of Nursing, Yonsei University, 50-1 Yonsei-ro, Seodamun-gu, Seoul, 03722 South Korea; 4 Psychology Department, College of Liberal Arts, Yonsei University, 50 Yonsei-ro, Seodamun-gu, Seoul, 03722 South Korea

**Keywords:** Hazard identification, Nursing, Nursing education research, Patient safety, Room of errors, Situation awareness

## Abstract

**Background:**

Patient safety is a critical concern in healthcare, with unsafe care causing significant harm. Nurses play a vital role in promoting safety and must be equipped with the skills to identify and manage safety hazards. The Room of Horrors (ROH) simulation was developed to enhance these skills by presenting learners with a simulated patient scenario containing safety hazards. This study aimed to evaluate the effectiveness of the simulation by (1) comparing hazard recognition performance across different groups; (2) assessing changes in self-perceived patient safety competency and confidence; (3) exploring participants’ simulation experiences; and (4) examining perceived benefits for clinical practice.

**Methods:**

A multi-method design was employed, incorporating a quasi-experimental three-group pre-test, post-test, and two-week follow-up structure, and a qualitative analysis of participants’ experiences and perceptions. The study involved participants from one nursing college and five hospitals in Korea. The sample (*N* = 90) comprised three groups: nursing students (*n* = 30), novice nurses (*n* = 30), and experienced nurses (*n* = 30). Participants underwent a 20-minute pre-briefing, 10-minute simulation, 10-minute self-reflection, and 40-minute debriefing session. Data were collected through structured surveys on patient safety competency, confidence, and open-ended questions about participants’ experiences and perceptions. Two-week follow-up surveys evaluated perceived clinical relevance. Quantitative data were analyzed using descriptive statistics and regression analysis; qualitative data were analyzed through content analysis.

**Results:**

Experienced nurses identified significantly more hazards, including those requiring two-step logical reasoning, than nursing students and novice nurses. Both novice and experienced nurses showed improvements in safety competency and confidence. Participant feedback was overwhelmingly positive, particularly highlighting the value of debriefing. The two-week follow-up indicated that almost all participants had applied the knowledge gained through the simulation in their clinical practice.

**Conclusions:**

The ROH simulation enhances self-reported patient safety competency and confidence, especially among experienced nurses, supporting its integration into nursing education and continuing professional development. Findings also suggest the importance of tailoring scenario complexity and debriefing strategies to learner readiness and highlight the potential value of integrating ROH simulations into experience-sensitive safety training programs. Further research is warranted to investigate its long-term impact on clinical practice.

**Trial registration:**

Not applicable.

**Supplementary Information:**

The online version contains supplementary material available at 10.1186/s12912-025-03700-x.

## Background

Unsafe care contributes significantly to patient harm and is a leading cause of mortality and morbidity worldwide [1]. In developed nations, approximately 10% of patients experience harm while receiving care, and patient adverse events account for 15% of overall hospital expenses in countries affiliated with the Organization for Economic Cooperation and Development [2]. Notably, nearly half of these events are considered preventable. During hospital stays, patients are exposed to various iatrogenic risks, such as inadequate hygiene practices and unsafe environments, which contribute to adverse events [3]. Although early identification and mitigation of hazards can prevent many of these events [4], such hazards frequently remain undetected and unaddressed in clinical settings [3].

Due to close contact with hospitalized patients, nurses play a vital role in promoting patient safety within hospital environments. Accordingly, both current and future members of the nursing workforce must be equipped with the knowledge and skills needed to identify safety risks and prevent hospital-acquired harm [5]. One innovative educational method for developing such skills is the “Room of Horrors (ROH)” simulation. This exercise involves a simulated patient room containing intentionally placed safety hazards. Learners are tasked with identifying and documenting these hazards within a clinical context [3, 6, 7]. The ROH simulation has been used to train a broad spectrum of healthcare professionals, including nursing students [8], medical students [9, 10], nurses [7, 11], interns [12, 13], and medical residents [9] in schools and hospitals in various countries.

Despite growing interest in ROH simulations as a patient safety training tool, a recent literature review [6] identified a lack of research involving nurses, who play a crucial role in recognizing and responding to safety hazards. Most existing studies have focused on descriptive outcomes (e.g., number of hazards identified) [9, 14–16], with limited exploration of learners’ perceived competency, confidence, or perceived application in clinical settings. Moreover, debriefing, which is an essential component of simulation-based education, has been applied inconsistently, with some studies incorporating structured discussions [11, 17], while others provided minimal or no opportunity for reflective learning [9, 14, 18]. These limitations make it difficult to evaluate the full educational impact of ROH simulations and to identify the best practices for their design and delivery.

A recent study employing the ROH simulation with diverse healthcare professionals in a U.S. hospital [19] found that although participants easily identified hazards associated with the presence of certain elements (e.g., two different wristbands), they often failed to identify hazards related to omission (e.g., no precaution sign) or those requiring two-step logical thinking (e.g., applying patient-controlled analgesia on unconscious patients). These findings are particularly relevant for nursing education, as nurses must be able to identify not only overt hazards but also those that are less obvious and more cognitively demanding. Previous studies have also noted differences in hazard identification between nursing students and nurses [11, 19]. Based on these findings, we hypothesized that differences in hazard recognition, particularly between obvious and cognitively complex hazards, would exist among nursing students, novice nurses, and experienced nurses, even when exposed to the same simulation scenario. Identifying such differences may help educators tailor ROH simulations to address the specific learning needs of each group, thereby improving the educational effectiveness of patient safety training. However, to our knowledge, no prior studies have examined these differences within the ROH simulation context.

This study addressed the following objectives: (1) to examine differences in hazard recognition among nursing students, novice nurses, and experienced nurses in order to inform the design of ROH simulations; (2) to assess the impact of the ROH simulation on participants’ self-assessed patient safety competency and confidence in identifying and managing hazards; (3) to explore participants’ experiences during the simulation; and (4) to investigate the benefits of the ROH simulation on clinical practice.

## Methods

### Study design

**Fig. 1:**
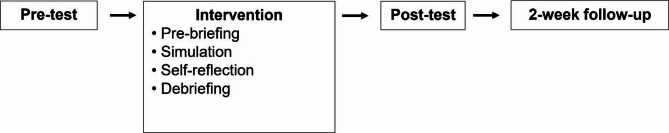
Research flow diagram

A multi-method design was employed. The study utilized a quasi-experimental, three-group pre-test, post-test, and two-week follow-up design [20] to evaluate the effectiveness of the ROH simulation (Fig. 1). In addition, qualitative data were collected at the post-test to explore participants’ experiences and perceptions.

### Study participants and setting

The study was conducted at a single simulation center in Korea. Participants included nursing students from one nursing college and nurses recruited from five hospitals. The study sample comprised three groups: (1) nursing students undergoing clinical practicum, (2) novice nurses with less than one year of nursing experience, and (3) experienced nurses with more than one year but less than five years of nursing experience. In Korea, nurses with fewer than 12 months of clinical experience are typically classified as novice nurses [21], while those with more than one year are considered experienced nurses [21]. Therefore, we recruited participants to align with the nursing student, novice nurse, and experienced nurse categories, using convenience sampling [8]. Notices were posted on the nursing college bulletin boards and online forums to recruit students, while nurses were recruited through coordination with each hospital’s nursing department. A priori power analysis using G*Power version 3.1.9.7 [22] indicated that a sample size of 90 (30 participants per group) would be required to detect a moderate to large effect size (*f* = 0.33 or partial $\:{\eta\:}^{2}$=0.1), with 0.8 power and a significance level of 0.05.

### The intervention

**Fig. 2:**
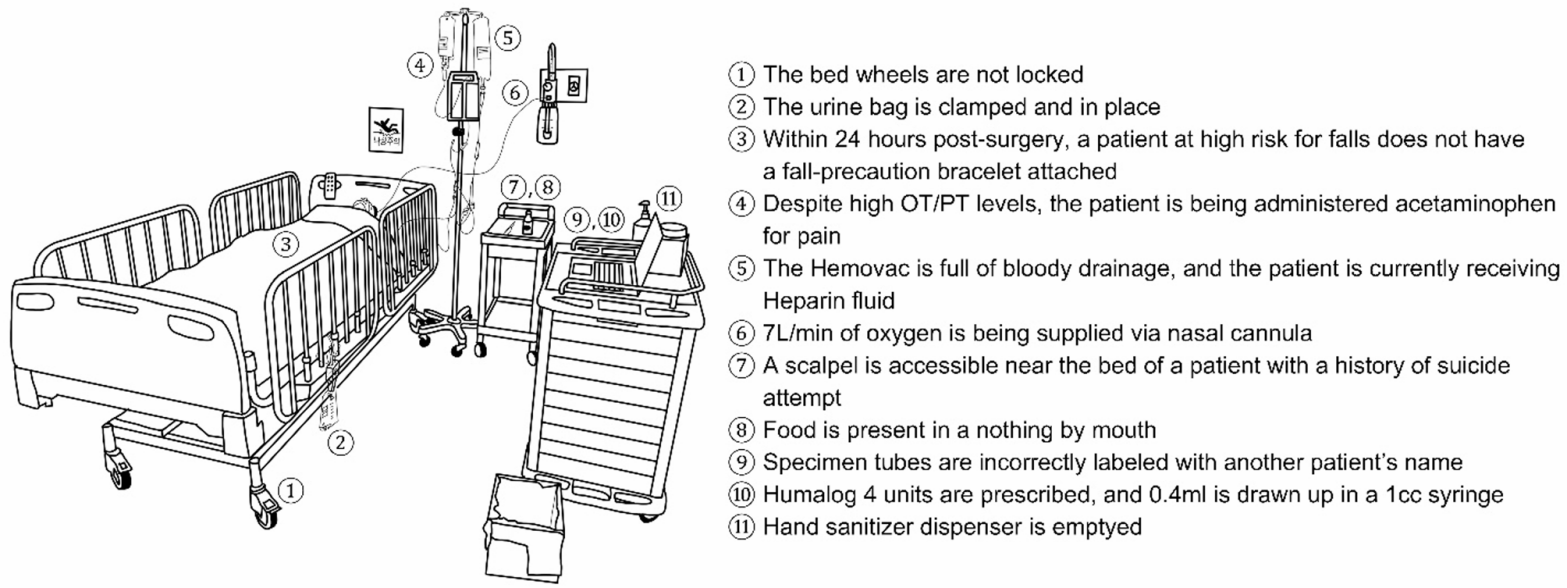
Landscape of the simulation room and a list of intentionally placed safety hazards. Note: OT = Aspartate aminotransferase. PT = Alanine aminotransferase

The intervention involved a simulated inpatient hospital room equipped with a case scenario, an electronic medical record (EMR), a mannequin, and 11 embedded safety hazards (Fig. 2). The clinical scenario was developed by a team of nurse researchers, patient safety experts, healthcare simulation experts, and nurse managers. The scenario featured a 72-year-old female patient with stomach cancer who had undergone a laparoscopic subtotal gastrectomy with gastroduodenostomy. The patient also had comorbid conditions, including type 2 diabetes mellitus, depression with a history of suicide attempt, and a previous cerebrovascular accident. The EMR included clinical data, physician orders, allergy information, a medication list, and laboratory results. The 11 safety hazards were aligned with the broad categories of the World Health Organization’s conceptual framework for the International Classification for Patient Safety [23]. As part of the scenario design, these hazards were pre-categorized into two types: six that were immediately observable (e.g., an unlocked bed or an empty sanitizer bottle) and six that required two-step logical reasoning (e.g., recognizing a medication error by comparing lab results and physician orders). This classification was embedded into the simulation structure to reflect varying levels of cognitive complexity and was applied consistently in subsequent analyses.

A structured 20-minute pre-briefing session was conducted to outline the learning objectives, participant expectations and responsibilities, the clinical context of the scenario, and logistical details [24]. A “fiction contract” was established to create a psychologically safe learning environment [25], in which participants agreed to engage fully, maintain confidentiality, and suspend disbelief during the simulation.

Each participant completed the simulation individually at a dedicated simulation center that was unfamiliar to them. Four rooms with identical patient scenarios and environmental layouts were used. Participants entered one of the rooms with a blank clipboard and were given 10 min to review the EMR and inspect the room for safety hazards. They documented identified hazards during this period before being instructed to exit by the simulation facilitators.

Following the simulation, participants completed a 10-minute self-reflection exercise guided by specific questions, encouraging them to reflect on how human and environmental factors influenced patient safety. This was followed by a 40-minute group debriefing session using the GAS (Gather, Analysis, Summarize) model [26], which supported structured reflection and guided discussion of the simulation experience. A total of 12 debriefing groups were conducted, each consisting of 6 to 8 participants, in accordance with recommended group sizes for effective debriefing [27, 28]. Nursing students were debriefed in groups composed exclusively of students, while novice and experienced nurses participated together in mixed groups, reflecting the reality of their interprofessional clinical environments. All debriefing sessions were conducted by a single faculty member who holds a certification in patient safety and error science and has completed formal training in both simulation-based education and structured debriefing methods. The use of a single qualified facilitator contributed to consistency in delivery across all debriefing sessions. Beyond supporting individual learning, these components were also intended to inform the development of more effective ROH simulations.

### Measures

All participants were asked to report demographic characteristics of age, gender, prior simulation experience (yes or no), and previous patient safety education (yes or no). Nursing students were also asked about their current year of study and prior hospital-related work experience. Novice and experienced nurses were asked about years of nursing experience, hospital tenure, and unit tenure.

Patient safety competency, defined as the knowledge, skills, and attitudes required to deliver safe patient care, was assessed using three subscales from the Health Professional Education in Patient Safety Survey [29]. This measure has demonstrated good reliability and validity with a Korean nurse sample [30]. The subscales included Managing Safety Risks (3 items), Recognize and Respond to Reduce Harm (2 items), and Understanding Human and Environmental Factors (2 items). These were selected by a team of nurse educators and patient safety experts for their relevance to the simulation. Each item was rated on a 5-point Likert scale ranging from 1 (*strongly disagree*) to 5 (*strongly agree*), with a higher mean score indicating a higher level of patient safety competency in the respective subscale. For the study sample, Cronbach’s alphas for the three subscales were 0.87, 0.82, and 0.86 for Time 1 and 0.87, 0.73, and 0.78 for Time 2, respectively.

Confidence in identifying and managing patient safety hazards was measured using two questions developed by the research team based on prior studies [12, 31]: “How confident are you in your ability to identify patient safety hazards? and “How confident are you in your ability to manage patient safety hazards?” Responses were rated on a 10-point scale ranging from 1 (*not at all confident*) to 10 (*extremely confident*).

Participants also evaluated the simulation experience using five items assessing perceived effectiveness in enhancing situational awareness, improving understanding of patient safety, acquiring new knowledge and skills, motivating patient safety improvement, and the usefulness of debriefing. Two additional items measured overall satisfaction and a willingness to recommend the intervention. Responses were rated on a 5-point Likert scale ranging from 1 (*strongly disagree*) to 5 (*strongly agree*). Three open-ended questions invited participants to describe positive aspects of the simulation, identify areas for improvement, and offer any additional comments.

Two weeks after the simulation, participants were asked to rate how helpful the intervention had been for their clinical practice using a 5-point response scale ranging from 1 (*not at all*) to 5 (*very much*), and to indicate whether they had applied the knowledge and skills learned (*yes/no*).

### Data collection

Data were collected at three time points: baseline (Time 1, before the pre-briefing session), immediately after the intervention (Time 2, after debriefing session), and two weeks post-intervention (Time 3). Online surveys were used for all data collection. Table 1 outlines the variables measured at each time point.

**Table 1:** Data collection

Pre-test	Post-test	2-week follow-up
• Demographic characteristics • Patient safety competency • Patient safety confidence	• Patient safety competency • Patient safety confidence • Effectiveness of ROH simulation • Participant experiences (open-ended questions)	• Perceived helpfulness • Practical application

Note: ROH = Room of horrors

### Statistical analysis

Descriptive statistics were used to summarize demographic characteristics, key study variables, and participants’ feedback on the ROH simulation. Participants’ performance in identifying patient safety hazards was assessed by the total number of correctly identified hazards out of 11. Analysis was conducted according to the two hazard categories that were pre-defined during scenario development: five requiring two-step logical reasoning and six that were immediately observable. Multiple regression analysis was used to compare the performance scores among the three groups, controlling for participants’ demographic characteristics and prior training experiences. Two dummy variables were created to represent the three groups, with experienced nurses as the reference category.

To evaluate group differences in changes in patient safety competency and confidence, multiple regression analyses were conducted on the difference scores between Time 1 and Time 2 (post-test minus pre-test), controlling for the same covariates. Difference score analysis was selected over ANCOVA due to its reported superiority in non-randomized designs [32]. All statistical analyses were performed using SPSS version 29, with a significance level of 0.05.

Qualitative data were used to supplement the quantitative findings. We followed conventional content analysis procedures as described by Hsieh and Shannon [33], which allows coding categories to emerge directly from the data without imposing a preconceived theoretical framework. Two researchers independently reviewed the narrative responses multiple times to become familiar with the content and identify meaningful units. Simple codes were assigned to each unit, and discrepancies were resolved through discussion. Recurring ideas were grouped into broad descriptive categories, and representative written comments were selected to illustrate participants’ perspectives.

## Results

### Participant characteristics and unadjusted group-level descriptive statistics

Most participants were female (92.2%), and the overall mean age was 25.12 years (*SD* = 2.49). The proportion of participants with prior simulation-based education was 56.7% for nursing students, 76.7% for novice nurses, and 73.3% for experienced nurses. Similarly, prior patient safety education had been received by 56.7% of students, 90.0% of novice nurses, and 86.7% of experienced nurses. On average, novice nurses had 4.10 months of clinical experience, whereas experienced nurses had 38.20 months (Table 2).

**Table 2:** Descriptive statistics by group (*N = 90*)

Variable	Nursing students (*n* = 30)	Novice nurses (*n* = 30)	Experienced nurses (*n* = 30)
	*N* (%) or Mean ± SD	*N* (%) or Mean ± SD	*N* (%) or Mean ± SD
Demographic characteristic			
Age (years)	22.93 ± 1.62	25.07 ± 1.82	27.37 ± 1.69
Gender			
Male	2 (6.7)	1 (3.3)	4 (13.3)
Female	28 (93.3)	29 (96.7)	26 (86.7)
Previous simulation-based education experience			
Yes	17 (56.7)	23 (76.7)	22 (73.3)
No	13 (43.3)	7 (23.3)	8 (26.7)
Previous patient safety education experience			
Yes	17 (56.7)	27 (90.0)	26 (86.7)
No	13 (43.3)	3 (10.0)	4 (13.3)
Grade^a^			
3rd	19 (63.3)	NA	NA
4th	11 (36.7)	NA	NA
Prior hospital-related work experience^a^			
Yes	12 (40.0)	NA	NA
No	18 (60.0)	NA	NA
Work unit^b^			
General unit	NA	23 (76.7)	22 (73.3)
Specialty unit	NA	7 (23.3)	8 (26.7)
Nursing experience^b^ (months)	NA	4.10 ± 3.80	38.20 ± 13.45
Hospital tenure^b^ (months)	NA	3.40 ± 3.29	35.83 ± 12.38
Unit tenure^b^ (months)	NA	3.27 ± 3.13	35.30 ± 11.72
Patient safety competency			
Managing safety risks			
Pretest score	2.96 ± 0.79	2.93 ± 0.63	3.52 ± 0.49
Posttest score	2.94 ± 0.57	3.06 ± 0.66	3.57 ± 0.60
Recognize and respond to reduce harm			
Pretest score	2.75 ± 0.87	2.73 ± 0.64	3.58 ± 0.48
Posttest score	2.97 ± 0.64	3.10 ± 0.66	3.63 ± 0.56
Understanding human and environmental factors			
Pretest score	3.70 ± 0.89	3.73 ± 0.74	4.00 ± 0.54
Posttest score	4.40 ± 0.53	4.20 ± 0.47	4.47 ± 0.57
Patient safety confidence			
Confidence in identifying safety risks			
Pretest score	5.33 ± 1.79	5.20 ± 1.47	6.53 ± 1.31
Posttest score	5.97 ± 1.50	5.97 ± 1.69	7.03 ± 1.30
Confidence in managing safety risks			
Pretest score	5.30 ± 1.93	4.90 ± 1.52	6.33 ± 1.16
Posttest score	5.70 ± 1.51	5.77 ± 1.68	6.87 ± 1.14
Room of Horror hazard identification			
Obvious safety hazards	2.13 ± 1.33	2.53 ± 1.01	3.23 ± 1.17
Two-step logical thinking hazards	1.00 ± 0.45	0.83 ± 0.65	1.30 ± 0.79

Notes: *SD* = Standard deviation; NA = Not applicable

^a^ Question asked to nursing students; ^b^ Question asked to nurses

As shown in Table 2, all groups showed improvement in patient safety competency and confidence scores from pre- to post-test. Within the competency subscales, nursing students showed the greatest improvement in ‘understanding human and environmental factors,’ with a mean increase of 0.70 points on a 5-point Likert scale. Based on the total of 11 embedded safety hazards, experienced nurses identified the highest number of hazards on average in both categories: two-step logical thinking hazards (*M* = 1.30 out of 5, *SD* = 0.79) and obvious hazards (*M* = 3.23 out of 6, *SD* = 1.17). However, it is important to note that the means presented in Table 2 are unadjusted descriptive comparisons across the three groups—nursing students, novice nurses, and experienced nurses. To control for potential confounding variables such as age, gender, and prior training, multiple regression analyses were subsequently conducted to estimate adjusted (conditional) means.

### Group differences in hazard identification

**Fig. 3:**
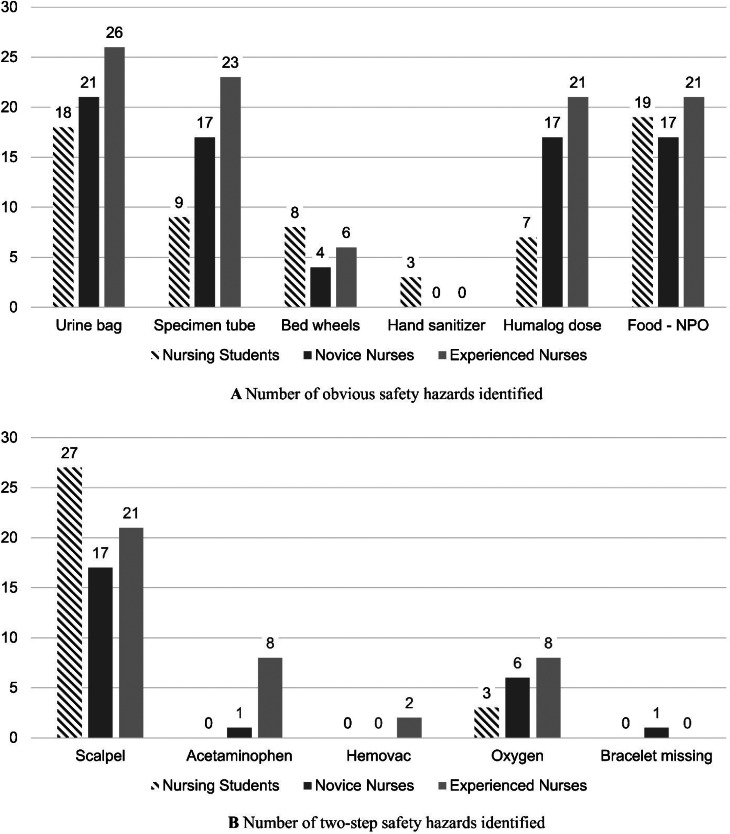
Hazard identification performance by hazard type and group. Notes: Bars represent the number of participants (out of 30) who correctly identified each hazard. Abbreviated labels are used for clarity. Full hazard descriptions: Urine bag = The urine bag is clamped and in place; Specimen tube = Specimen tubes are incorrectly labeled with another patient’s name; Bed wheels = The bed wheels are not locked; Hand sanitizer = The hand sanitizer dispenser is empty; Humalog dose = Humalog 4 units are prescribed, but 0.4 ml is drawn up in a 1 cc syringe; Food-NPO = Food is present despite the “nothing by mouth” order; Scalpel = A scalpel is accessible near the bed of a patient with a history of suicide attempt; Acetaminophen = Despite high AST/ALT levels, the patient is being administered acetaminophen for pain; Hemovac = The hemovac is full of bloody drainage, and the patient is currently receiving Heparin fluid; Oxygen = 7 L/min of oxygen is being supplied via nasal cannula; Bracelet missing = Within 24 h post-surgery, the patient at high risk for falls does not have a fall-precaution bracelet attached

Figure 3 presents the number of participants in each group who correctly identified the 11 safety hazards embedded in the ROH simulation. Each bar reflects the number of participants (out of 30 in each group) who recognized each hazard. More obvious hazards, such as the clamped urine bag and the presence of food despite a “nothing by mouth” order, were recognized by a majority of participants. In contrast, complex hazards requiring two-step reasoning, such as the continued administration of acetaminophen despite elevated liver enzymes or the presence of a full Hemovac in a patient receiving heparin, were frequently overlooked. These findings indicate that while certain overt hazards are readily detected, cognitively demanding scenarios may be more challenging for learners to identify.

In addition to item-level frequencies, adjusted mean scores for the number of hazards correctly identified per participant were estimated using multiple regression analysis. These models controlled for age, gender, and prior training experience, with estimates based on a reference participant profile (female, 25.12 years old, no prior simulation or patient safety education). This approach allows for fair comparisons across the groups by holding constant individual-level covariates that could affect performance. Results indicated that experienced nurses identified significantly more obvious hazards (adjusted *M* = 3.35 out of 6) than novice nurses (*M* = 2.60) and nursing students (*M* = 2.17). A similar pattern was observed for two-step logical reasoning hazards (maximum = 5), where experienced nurses (*M* = 1.59) outperformed both novice nurses (*M* = 0.99) and students (*M* = 1.05). No significant difference was observed between nursing students and novice nurses in either hazard category.

These findings underscore the importance of clinical experience in hazard recognition. Even when individual characteristics are held constant, experienced nurses demonstrated a greater ability to identify both overt and cognitively complex safety hazards, supporting the need for experience-sensitive simulation design. Full regression coefficients and confidence intervals are provided in Supplementary Table S1.

### Adjusted pre-post changes in patient safety competency and confidence by group

Table 3 shows the adjusted pre-post difference scores for the three patient safety competency subscales and confidence measures across the three groups. All values were adjusted using multiple regression, controlling for age, gender, and prior training, based on a reference participant profile (female, age 25.12, no prior simulation or safety training).

For ‘Managing Safety Risks,’ both novice (adjusted pre–post difference = 0.45) and experienced nurses showed the highest improvement (adjusted pre–post difference = 0.58), while no significant change was observed for students. For ‘Recognize and Respond to Reduce Harm,’ only novice nurses demonstrated a significant gain (adjusted pre–post difference = 0.54). In contrast, all three groups showed significant improvements in ‘Understanding Human and Environmental Factors’: students (adjusted pre–post difference = 0.77), novice nurses (adjusted pre–post difference = 0.57), and experienced nurses (adjusted pre–post difference = 0.57).

Regarding confidence in identifying patient safety hazards, novice (adjusted pre–post difference = 1.35) and experienced nurses (adjusted pre–post difference = 1.56) showed significant improvements, while nursing students did not. For confidence in managing safety hazards, only experienced nurses reported a significant increase (adjusted pre–post difference = 1.13). Full regression results are provided in Supplementary Table S2.

**Table 3:** Adjusted pre–post difference scores for patient safety competency and confidence by group (*N* = 90)

Variable	Adjusted mean difference (post – pre) (95% CI)		
	Nursing students (*n* = 30)	Novice nurses (*n* = 30)	Experienced nurses (*n* = 30)
Managing safety risks	0.04^a^(-0.37, 0.45)	0.45*^b^ (0.02, 0.88)	0.58*^b^ (0.13, 1.02)
Recognize and respond to reduce harm	0.21^a^ (-0.24, 0.65)	0.54*^a^ (0.07, 1.00)	0.44^a^ (-0.04, 0.92)
Understanding human and environmental factors	0.77*^a^ (0.30, 1.24)	0.57*^a^ (0.08, 1.06)	0.57*^a^ (0.06, 1.08)
Confidence in identifying patient safety hazards	0.64^a^ (-0.40, 1.67)	1.35*^a^ (0.26, 2.43)	1.56*^a^ (0.43, 2.68)
Confidence in managing patient safety hazards	0.02^a^ (-0.93, 0.97)	0.91^a^ (-0.08, 1.91)	1.13*^a^ (0.10, 2.16)

Note: CI = Confidence interval

Adjusted mean difference scores were estimated using multiple regression models, based on a reference participant (female, 25.12 years old, no prior simulation or patient safety training). Positive values indicate an increase from pre- to post-test. Significant changes in scores are denoted by an asterisk (*) symbol

Superscripts: Means sharing the same letter are not significantly different (p ≥ .05). Means with different letters differ significantly (p < .05)

### Post-simulation evaluation of the simulation and 2-week follow-up on clinical application

Immediately following the ROH simulation, participants provided overwhelmingly positive evaluations. Between 97% and 100% agreed or strongly agreed that the intervention was highly effective in enhancing the perceived importance of situational awareness, patient safety understanding, knowledge acquisition, and motivation for patient safety improvements. Additionally, participants found the debriefing sessions particularly beneficial for consolidating their learning. Overall satisfaction with the simulation was high, and most participants indicated they would recommend the program to others. In a follow-up survey conducted two weeks after the intervention, all but two participants reported that the simulation had been helpful in their clinical practice. Most also indicated that they had applied the knowledge gained during the session (See Supplementary Table S3).

### Qualitative findings from open-ended responses

Participants provided detailed feedback through open-ended questions, describing helpful aspects of the simulation program, areas for improvement, and additional comments or suggestions. This qualitative feedback captured a comprehensive view of their experiences and insights. All participants responded to each question, and their responses revealed six key themes. The findings are discussed below, with exemplary comments presented in Table 4.

**Table 4:** The results of qualitative data analysis (*N = 90*)

Theme	Written comment
Enjoyable, realistic, and applied learning	• Being able to apply what I learned in the classroom to realistic situations would really help me remember the lessons. (Nursing student, 26)
	• Experiencing nursing in situations similar to what I had only imagined really made it resonate with me. I feel like I’ve grown from the experience. (Novice nurse, 41)
	• It was fun finding errors in a setting that felt like a real situation, and I think I can apply what I learned to my clinical practice (Experienced nurse, 65)
Psychologically safe and supportive educational atmosphere	• I liked that we weren’t being evaluated and could participate comfortably… (continued)… I also appreciated the debriefing sessions where we could freely share our opinions. (Nursing student, 8)
	• It was not just enjoyable, but I also appreciated learning various knowledge and perspectives on patient safety in a psychologically comfortable environment. (Novice nurse, 89)
	• I appreciated the pressure-free environment. (Experienced nurse, 21)
Reflective practice and professional growth	• Through this program, I have developed a sense of awareness about the importance of carefully examining the patient’s environment. (Nursing student, 4)
	• It was a motivating time that made me realize the importance of paying more attention to patient safety. (Novice nurse, 43)
	• I have never really paid attention to potential errors when assessing patients previously, but I think I will check more thoroughly for any risk factors in patients during my shift tomorrow. (Experienced nurse, 22)
Need for wider implementation	• It would be great to have these kinds of simulations included in the school curriculum. (Nursing student, 16)
	• I hope there will be more of these patient safety education programs. (Novice nurse, 87)
	• It would be beneficial to have these kinds of programs as part of hospital’s nurse training programs. (Experienced nurse, 76)
Recommendations for future interventions	• I wish we had more time for simulation. (Novice nurse, 46)
	• The debriefing was incredibly valuable. I believe an extended session would enhance the learning experience even further. (Novice nurse, 86)

#### Theme one: engaging, realistic, and applied learning

The simulation offered an engaging and immersive environment that closely resembled real clinical settings, allowing participants to apply theoretical knowledge in practical scenarios. This experience enhanced their understanding and retention of key concepts. Participants noted that the enjoyable and realistic format helped bridge the gap between theory and practice. They also noted that the inclusion of both obvious hazards and those requiring two-step logical thinking made the learning more beneficial. Overall, participants viewed the simulation as more impactful than traditional learning methods.

#### Theme two: psychologically safe and supportive educational atmosphere

Participants highlighted that the psychologically safe, non-evaluative atmosphere reduced the stress often associated with traditional clinical training, allowing them to focus more fully on learning. They valued the opportunity to take risks and make mistakes without the pressure of being graded, which fostered open discussion and promoted deeper understanding during the simulation and debriefing sessions.

#### Theme three: reflective practice and professional growth

Participants emphasized that the debriefing sessions were essential for reinforcing learning, promoting reflection, and supporting professional development. These sessions allowed them to critically evaluate their actions, share insights, and explore missed safety hazards. Reflective learning during debriefing enhanced their critical thinking and observational skills.

This process encouraged participants to recognize knowledge gaps, increase their awareness of patient safety, and feel a stronger sense of responsibility in clinical settings. Participants reported that the simulation was not only educational but also motivational, inspiring them to be more meticulous in patient care and more proactive in identifying safety risks. Many expressed a renewed commitment to applying these insights in their clinical practice.

Novice nurses described the simulation as a motivational experience that heightened their vigilance in practice and encouraged them to review and apply what they had learned. Experienced nurses reported that the intervention prompted them to reevaluate their own assumptions and renew their focus on fundamental safety principles. Some acknowledged previously overlooked risks in their work environment and emphasized the importance of reinforcing basic safety practices.

#### Theme four: need for wider implementation

Many participants expressed strong support for broader integration of similar simulation programs into both basic and continuing nursing education, emphasizing their relevance across all career stages. Nursing students highlighted the potential value of incorporating this type of training into various subject areas within their curriculum. Novice and experienced nurses alike viewed the intervention as a meaningful addition to continuing education, such as hospital orientation or in-service programs, noting its potential to reduce future errors and enhance patient safety.

#### Theme five: recommendations for future interventions

When asked about areas for improvement, participants commonly recommended allocating more time for both the simulation and debriefing sessions. They emphasized the importance of debriefing, noting that they gained significant insights during these discussions and would benefit from an extended debriefing period.

## Discussion

This study examined the effectiveness of the ROH simulation in enhancing patient safety competency and confidence in identifying and managing safety hazards among nursing students, novice nurses, and experienced nurses. It also assessed participants’ abilities to identify safety hazards embedded in the simulation scenario. Our findings align with previous studies reporting that ROH simulations can enhance safety-related knowledge [18] and confidence in hazard identification [12] and management [34]. Significant post-simulation improvements in patient safety competency and confidence were observed across all groups, although the extent varied by clinical experience. Notably, both novice and experienced nurses demonstrated significant gains, suggesting that the ROH simulation may be particularly effective for individuals with prior clinical experience, possibly due to their foundational knowledge and practical exposure [3].

A consistent challenge identified in our study and Wang et al. [7] was the difficulty participants had in identifying complex hazards, particularly those involving omissions or requiring two-step logical thinking. This suggests a universal difficulty across clinical roles and settings. Our results further showed that experienced nurses significantly outperformed novice nurses and students in identifying both obvious and complex hazards, reinforcing the idea that clinical experience enhances hazard recognition [35, 36]. This aligns with previous studies involving medical students and professionals [9, 37], which also highlight variability in hazard identification. These findings support the view that while ROH simulations are effective, their impact is strengthened when paired with real-world clinical practice.

Consistent with previous studies emphasizing the educational value of simulation-based training [3, 10, 19], participants in our study gave highly positive feedback on the ROH experience. They appreciated the realistic and interactive nature, noting that it effectively bridged the gap between theoretical knowledge and practical application, thereby enhancing their comprehension and retention of patient safety principles. The two-week follow-up survey suggested the potential for sustained impact, as most participants reported applying the acquired knowledge and skills in clinical settings. However, further longitudinal studies are needed to confirm longer term effects. In line with prior findings [38], our results indicate that ROH simulations not only improve immediate learning outcomes but may also contribute to sustained improvements in patient safety practices. However, this finding alone is insufficient to determine its long-term impact. Future longitudinal studies with extended follow-ups and objective patient outcome measures are necessary to evaluate the lasting effects of such interventions on outcomes [39, 40].

Debriefing sessions emerged as a critical component in reinforcing learning. As emphasized by Zimmermann et al. [3], high-quality debriefing immediately following simulation enhances participants’ ability to reflect on their performance and grasp the implications of the identified hazards. This aligns with the qualitative feedback from our participants, who valued the opportunity to discuss their experiences, learn from mistakes, and exchange diverse perspectives in a supportive environment. Consistent with prior research on high-fidelity [41–43] and virtual reality-based simulations [44, 45], our findings reinforce the role of debriefing in promoting reflection and deeper learning. These results underscore the necessity of incorporating structured debriefing into ROH simulation programs to optimize their educational impact.

Open-ended feedback revealed that the effects of debriefing varied by participants’ experience. Nursing students reported that debriefing helped them recall and contextualize textbook knowledge, effectively bridging the gap between theory with practice. Novice nurses noted gaining insight into their clinical shortcomings through peer discussions and valued the opportunity to learn diverse perspectives and strategies for improving patient safety. Experienced nurses described engaging in deeper reflection during debriefing, which facilitated knowledge exchange and prompted reflection on aspects of nursing care that are often overlooked in daily practice. They also proposed concrete plans to apply their learning in practice. These insights suggest that tailored debriefing approaches may further enhance learning for different experience levels [46, 47]. Our findings have implications for the instructional design of ROH simulations. Scenarios should include a balance of both obvious and cognitively complex hazards to evaluate the full spectrum of learners’ hazard recognition abilities [7]. Unlike previous ROH studies, which often reported only the total number of hazards identified, our study differentiated between types of hazards and revealed how recognition patterns varied by participants’ clinical experience. This approach allowed us to uncover meaningful differences in how learners at different stages process and respond to safety cues. To facilitate deeper reflection and promote knowledge retention, debriefing strategies should be aligned with learner readiness and experience levels [45, 46]. Tailoring both the complexity of hazards and the structure of debriefing accordingly may enhance educational outcomes. Our findings highlight the potential value of integrating ROH simulations into a longitudinal learning model, starting in pre-clinical education and continuing into clinical practice through follow-up training and reflective debriefing. Given the observed differences across experience levels, such a staged approach may better support the development of hazard recognition skills over time.

This study has several limitations. First, while the study integrates both quantitative and qualitative data, the non-randomized design limits the ability to draw definitive causal conclusions about the effects of the ROH simulation on the observed improvements in patient safety competency and confidence. Second, the absence of a control group restricts direct comparisons between the intervention and standard training or no intervention. Controlled trials could examine how ROH simulation compares to other instructional approaches in improving hazard identification and patient safety outcomes. Third, although the sample size was adequate for statistical analysis, it may not fully represent the broader nursing population. Convenience sampling may have introduced selection bias, as participants could have had a pre-existing interest in patient safety. Although differences in participant reflections were observed across experience levels, it is unclear whether these were influenced by the composition of the debriefing groups. Nonetheless, future studies could examine how homogeneous versus mixed group debriefing sessions may shape the nature of reflection and discussion. Moreover, our instructional design incorporated both individual reflection and group debriefing components: participants first engaged in a structured self-reflection exercise, followed by a facilitated group discussion. This two-stage approach appeared to support deeper learning, and future research may explore its comparative benefits in more detail. Lastly, the two-week follow-up period provides only a short-term perspective. Longer-term studies with extended follow-up and objective outcome measures are necessary to assess the sustained impact of ROH simulations on clinical practice and patient safety outcomes.

## Conclusion

Patient safety remains a critical concern in healthcare, requiring nurses to develop competencies in identifying and preventing harm. This study suggests that the ROH simulation may be an effective educational tool for enhancing patient safety competency among nursing students, novice nurses, and experienced nurses. While all groups benefited, the degree of impact varied according to prior clinical experience, suggesting the need for tailored educational strategies. Participants’ positive feedback highlighted the value of engaging, realistic, and supportive learning environments, as well as the pivotal role of debriefing in consolidating learning outcomes. By integrating both quantitative and qualitative findings, this study provides a comprehensive understanding of the participants’ experiences and the effectiveness of the ROH simulation. Incorporating ROH simulations into nursing education and continuing professional development may enhance nurses’ preparedness and contribute to safer clinical practice. However, further research is needed to examine long-erm effects and optimize its implementation across diverse educational and clinical contexts.

## Supplementary Information

Below is the link to the electronic supplementary material.

Supplementary Material 1

## Data Availability

The datasets generated and/or analysed during the current study are not publicly available as they contain information that could compromise the consent of research participants. However, they can be provided by the corresponding author upon reasonable request.

## Abbreviations

EMR: Electronic medical record
ROH: Room of horrors
