# Supplementary material for: Patient safety in the ‘Room of Horrors’ simulation: a multi-method study of student, novice, and experienced nurses
Source: BMC Nurs. 2025 Aug 8;24:1044. doi: 10.1186/s12912-025-03700-x (PMC12335060; doi:10.1186/s12912-025-03700-x)
Supplement: Supplementary file 1 — Supplementary Material 1 [file 12912_2025_3700_MOESM1_ESM.docx]

**Supplementary Material 1**

**Description:**

Table S1, S2: Ordinary least squares (OLS) regression was used to compare the performance scores among the three groups, controlling for demographic variables and participants’ previous educational experiences. OLS regression was conducted using SPSS version 29 at a significance level of 0.05.

Table S3: Participants' evaluation of the ROH simulation was gathered immediately after the simulation and two weeks following the simulation.

**Table S1** Regression on number of identified obvious and two-step logical thinking safety hazards

|  | Outcomes | |
| --- | --- | --- |
| Regression Coefficients | Number of identified obvious safety hazards | Number of identified hazards that require two-step logical thinking |
| Intercept: experienced nurses | 3.352 (0.389)  *p* < .001 | 1.586 (0.209)  *p* < .001 |
| Nursing students dummy* | -1.179 (0.454)  *p* = .011 | -0.533 (0.245)  *p* = .032 |
| Novice nurses dummy* | -0.755 (0.351)  *p* = .034 | -0.596 (0.189)  *p* = .002 |
| Age (mean-centered) | 0.001 (0.077)  *p* = .989 | -0.040 (0.041)  *p* = .342 |
| Male (0=female;  1=male) | -0.576 (0.488)  *p* = .241 | -0.449 (0.263)  *p* = .092 |
| Simulation training experience | 0.348 (0.313)  *p* = .269 | -0.230 (0.169)  *p* = .176 |
| Patient safety experience | -0.345 (0.369)  *p* = .351 | 0.036 (0.199)  *p* = .855 |

* Reference group is experienced nurses. Contact corresponding author for regression results

using different reference groups.

**Table S2** Regression of patient safety competency and confidence pre- and post-test difference scores

| Outcomes (posttest – pretest scores) | | | | | |
| --- | --- | --- | --- | --- | --- |
| Regression coefficients | Managing safety risks | Recognize and respond to reduce harm | Understanding human and environment factors | Confidence in identifying patient safety hazards | Confidence in managing patient safety hazards |
| Intercept: experienced nurses | 0.577 (0.224)  *p* = .012 | 0.439 (0.242)  *p* = .073 | 0.573 (0.256)  *p* = .028 | 1.555 (0.564)  *p* =.007 | 1.132 (0.518)  *p* =.032 |
| Nursing students dummy* | -0.537 (0.262)  *p* = .043 | -0.234 (0.282)  *p* = .409 | 0.194 (0.300)  *p* = .518 | -0.916 (0.659)  *p* =.168 | -1.114 (0.606)  *p* =.070 |
| Novice nurses dummy* | -0.123 (0.202)  *p* = .543 | 0.098 (0.218)  *p* = .656 | -0.002 (0.232)  *p* = .993 | -0.209 (0.509)  *p* =.682 | -0.218 (0.468)  *p* =.643 |
| Age (mean-centered) | -0.088 (0.044)  *p* = .049 | -0.078 (0.048)  *p* = .107 | -0.008 (0.051)  *p* = .877 | -0.199 (0.112)  *p* =.079 | -0.229 (0.103)  *p* =.029 |
| Male (0=female;  1=male) | -0.118 (0.281)  *p* = .675 | -0.472 (0.303)  *p* = .123 | 0.106 (0.322)  *p* = .742 | -0.426 (0.708)  *p* =.549 | -0.310 (0.651)  *p* =.635 |
| Simulation training experience | -0.323 (0.180)  *p* = .077 | -0.330 (0.194)  *p* = .094 | -0.278 (0.206)  *p* = .181 | -0.593 (0.454)  *p* =.195 | -0.783 (0.417)  *p* =.064 |
| Patient safety experience | -0.094 (0.212)  *p* = .659 | 0.105 (0.229)  *p* = .649 | 0.117 (0.243)  *p* = .631 | -0.134 (-.535)  *p* =.802 | 0.612 (0.492)  *p* =.217 |

* Reference group is experienced nurses. Contact corresponding author for regression results using different reference groups.

**Table S3** Participants feedback on the Room of Horror simulation and its preliminary long-term effect (*N* = 90)

| Questions | Nursing Students  (*n* = 30) | Novice Nurses  (*n* = 30) | Experienced Nurses  (*n* = 30) |
| --- | --- | --- | --- |
| **Immediate after the intervention** |  |  |  |
| Enhancing situational awareness |  |  |  |
| Strongly agree | 24 (80.0%) | 23(76.7%) | 16 (53.3%) |
| Agree | 6 (20.0%) | 7 (23.3%) | 14 (46.7%) |
| Neutral, disagree, or strongly disagree | ---------------------------None------------------------ | | |
| Improved understanding of patient safety |  |  |  |
| Strongly agree | 21 (70%) | 19 (63.3%) | 19 (63.3%) |
| Agree | 8 (26.7%) | 11 (36.7%) | 11 (36.7%) |
| Neutral | 1 (3.3%) | 0 (0.0%) | 0 (0.0%) |
| Disagree or strongly disagree | ---------------------------None------------------------ | | |
| Acquisition of new knowledge and skills for safe care |  |  |  |
| Strongly agree | 19 (63.3%) | 18 (60.0%) | 16 (53.3%) |
| Agree | 11 (36.7%) | 12 (40.0%) | 14 (46.7%) |
| Neutral, disagree, or strongly disagree | ---------------------------None------------------------ | | |
| Motivation for improving patient safety |  |  |  |
| Strongly agree | 25 (83.3%) | 20 (66.7%) | 20 (66.7%) |
| Agree | 5 (16.7%) | 10 (33.3%) | 10 (33.3%) |
| Neutral, disagree, or strongly disagree | ---------------------------None------------------------ | | |
| Utility of debriefing for learning |  |  |  |
| Strongly agree | 28 (93.3%) | 23 (76.7%) | 26 (86.7%) |
| Agree | 2 (6.7%) | 7 (23.3%) | 4 (13.3%) |
| Neutral, disagree, and strongly disagree | ---------------------------None------------------------ | | |
| Overall satisfaction with the simulation program |  |  |  |
| Strongly agree | 27 (90.0%) | 19 (63.3%) | 24 (80.0%) |
| Agree | 3 (10.0%) | 11 (36.7%) | 6 (20.0%) |
| Neutral, disagree, or strongly disagree | ---------------------------None------------------------ | | |
| Likelihood of recommending the program to others |  |  |  |
| Strongly agree | 29 (96.7%) | 18 (60.0%) | 23 (76.7%) |
| Agree | 1 (3.3%) | 11 (36.7%) | 7 (23.3%) |
| Neutral | 0 (0.0%) | 1 (3.3%) | 0 (0.0%) |
| Disagree or strongly disagree | ---------------------------None------------------------ | | |
| **Two-weeks after the intervention** |  |  |  |
| Helpfulness of the program for your clinical practices |  |  |  |
| Strongly agree | 20 (66.7%) | 14 (46.7%) | 11 (36.7%) |
| Agree | 10 (33.3%) | 15 (50.0%) | 18 (60.0%) |
| Neutral | 0 (0.0%) | 1 (3.3%) | 1 (3.3%) |
| Disagree or strongly disagree | ---------------------------None------------------------ | | |
| Application of knowledge from program to clinical practice |  |  |  |
| Yes | 29 (96.7%) | 28 (93.3 %) | 30 (100.0 %) |
| No | 1 (3.3%) | 2 (6.7%) | 0 (0.0%) |
